# Effects of Ashwagandha (*Withania somnifera*) on Body Composition: A Narrative Review

**DOI:** 10.3390/sports14100442

**Published:** 2026-10-09

**Authors:** Giuliet L. Castropa, Nathaniel Rhoades, Yuhang Liu, Ian H. Bivins, Drew E. Gonzalez, Richard B. Kreider

**Affiliations:** 1 Exercise & Sport Nutrition Laboratory, Department of Health and Kinesiology, College Station, TX 77843, USA; ndrhoades@tamu.edu (N.R.); yhliu@tamu.edu (Y.L.); ian.bivins@tamu.edu (I.H.B.); drewgonzalez418@shsu.edu (D.E.G.); rbkreider@tamu.edu (R.B.K.); 2 Occupational, Performance, and Nutrition Laboratory, Department of Kinesiology, College of Health Sciences, Huntsville, TX 77340, USA

**Keywords:** herbal supplements, adaptogen, body mass index, fat mass, muscle mass, waist circumference

## Abstract

Ashwagandha (*Withania somnifera*) has gained interest as an anti-stress agent due to its withanolide content. Ashwagandha’s adaptogenic nature, through regulation of the hypothalamic–pituitary–adrenal (HPA) axis, makes it a promising agent for improving responses to external stressors, including changes in body composition. Studies have demonstrated that ashwagandha can positively affect stress regulation, metabolism, and muscle strength, but how these adaptations impact body composition has not been thoroughly described. Theoretically, ashwagandha’s mechanistic effects may reduce body fat and increase muscle mass, thereby optimizing body composition. This review summarizes the current evidence on the effects of ashwagandha supplementation on body composition metrics and proposes mechanisms that explain the results. Therefore, this literature review analyzed the current literature examining changes in body composition metrics, including body mass, fat mass, lean mass, muscle mass, and anthropometric measurements, in adults when supplementing with ashwagandha. The discussion details specific protocol and population differences between the included studies and how these differences influence body composition results. The discussion also outlines future research directions on potential effects on weight management and sex-specific adaptation to better understand ashwagandha’s underlying effects on body composition.

## 1. Introduction

Ashwagandha (*Withania somnifera*) is a natural herb originating from India and used in traditional Ayurveda medicine. The primary bioactive compounds in ashwagandha (ASH) are the withanolides, naturally occurring polyoxygenated steroidal lactones. ASH is generally regarded as an adaptogen, referring to a class of compounds proposed to enhance nonspecific resistance to physical, chemical, and biological stressors [1,2]. Among ASH’s proposed adaptogenic properties, stress reduction has received the greatest scientific attention. Current evidence suggests that ASH may exert these effects by modulating the hypothalamic–pituitary–adrenal (HPA) axis. Hyperactivity of the HPA axis increases stress by increasing cortisol, adrenaline, and norepinephrine, which underlie psychiatric disorders like depression, anxiety, and sleep disorders [3]. ASH supplementation is associated with lower levels of morning cortisol [4,5], with well-supported reductions in stress, depression, and anxiety, and improvements in insomnia symptoms [6]. Regulation of the HPA axis may improve glucose homeostasis, reduce the catabolic effects of stress [3,7], and even lead to healthier eating habits, including reduced consumption of hedonic, energy-dense foods [8]. Therefore, ASH’s potential to improve stress regulation may represent one pathway through which it could mitigate metabolic dysregulation.

Excess body weight is a major global public health concern, with rates of overweight and obese individuals rising [9]. The obesity epidemic in the United States is well-documented, with the rates of obesity continuing to rise [10,11,12,13]. The CDC estimates that 41.9% of United States adults are classified as obese, with a body mass index (BMI) over 30 kg/m^2^ [14]. Current projections further suggest that nearly half of U.S. adults may be living with obesity by 2030 [15]. Excess body weight is associated with many comorbidities, including hypertension, coronary artery disease, type 2 diabetes, metabolic syndrome, osteoarthritis, nonalcoholic fatty liver disease, chronic kidney disease [16], and all-cause mortality [17]. Given the severity of health problems associated with excess body weight, it is important to seek practical weight-management methods. ASH may improve body composition by inhibiting adipogenesis [18], improving glucose sensitivity, regulating adipocytokine activity, enhancing lipid metabolism [19], and controlling eating patterns through stress modulation [8]. Collectively, these effects may support weight management and metabolic health in individuals with obesity.

Athletes benefit from optimized body composition, including performance improvements. Generally, athletes have higher relative lean mass than the general population [20]. However, optimal body composition heavily depends on the type of exercise and whether it is for performance or aesthetic reasons. For example, endurance athletes and bodybuilders may benefit from increased lean mass and reduced fat mass during the competition season [21,22]. In contrast, some power and high-contact athletes focus on maximizing overall body mass relative to effective performance [23]. Athletes may be tempted to perform extreme diets to reach their optimal body composition, but this strategy can damage performance and increase the risk of injury or illness [24]. Thus, athletes also need pragmatic methods to improve their body composition to enhance sports performance and overall health. A primary adaptogenic mechanism of ASH supplementation in response to physical stressors may be its antioxidant and anti-inflammatory properties, particularly those of withaferin A. This withanolide suppresses NF-κB in vitro [25], induces the transcription factor Nrf2 in mice [26], and induces antioxidant expression in cardiomyocytes under simulated ischemic reperfusion injury [27]. In theory, the antioxidant perfusion effects would help deliver nutrients to exercising muscle, and anti-inflammatory properties may reduce the negative pain effects of exercise, leading to improved performance [28]. Therefore, ASH’s withanolide concentration may enable those undergoing a training program to tolerate greater loads by delaying fatigue [29], promoting greater muscle adaptation through its antioxidant and anti-inflammatory properties. Additionally, ASH has been shown to increase serum and salivary testosterone and DHEA-S levels in men [4,30,31,32,33]. As DHEA-S and testosterone are vital regulators of muscle growth [34], athletes benefit from increased concentrations of anabolic steroid hormones. Thus, ASH appears promising for increasing muscle mass, also through natural improvements in hormone profiles.

Given its antioxidant, stress-reducing, hormone-modulating, and potential weight-management properties, ASH supplementation may improve body composition. However, to date, there is no comprehensive review on the effects of ASH supplementation on changes in body composition metrics. In a systematic review of performance adaptations with ASH, Bonilla et al. [2] identified the effects of ASH on body composition as a future research direction. Since that review, research assessing ASH’s effect on body composition has increased and warrants a more comprehensive review of the literature. Accordingly, this review aims to evaluate the effect of ashwagandha (*Withania somnifera*) supplementation on body composition measures, including body mass, fat mass, lean mass, muscle mass, and anthropometric circumferences, and to further examine its potential combined effects with dietary and exercise interventions.

## 2. Materials and Methods

A narrative review with a systematic approach to searching the literature was conducted between 20–23 February 2026, using PubMed (National Center for Biotechnology Information, U.S. National Library of Medicine; URL: https://pubmed.ncbi.nlm.nih.gov/, accessed on 23 February 2026), Google Scholar (Google LLC; URL: https://scholar.google.com, accessed on 23 February 2026), and Web of Science (Clarivate; URL: webofscience.com, accessed on 23 February 2026) databases to identify clinical studies examining the effects of ASH on various body composition measures in adults.

Studies were included if they examined oral ASH supplementation in adult humans with a placebo (PLA) comparison and reported body composition outcomes in healthy, overweight, obese, or athletic populations. The search strategy included the following terms and Boolean operators: “*Withania somnifera*” OR “Ashwagandha” AND “Anthropometry”, “Body Composition”, “Body Fat”, Body Water”, “Bone Density”, “Bone Mineral”, “Bone Mineral Content”, “Fat”, “Fat-Free”, “Lean Mass”, “Muscle”, “Muscle Mass”, “Muscle Tissue”, “Circumference”, OR “Waist Circumference”. Studies using multi-ingredient supplements were considered if they included a true PLA for ASH. Studies focusing on disease populations and animal or in vitro experiments were excluded. Studies were excluded if they were unavailable in English, did not report body composition metrics before and after supplementation, or did not provide original data. After initial screening, full texts of potentially relevant articles were reviewed. Additional clinical trials meeting these criteria were identified through citations of these previously identified studies. Relevant results were extracted and summarized for review.

## 3. Results

A total of 16 studies were included based on the search method described above (Table 1). Four of these studies were sponsored by commercial funding as indicated in Table 1. This section describes evidence on the effects of ASH supplementation on body composition measures, including body mass, fat mass, lean mass, muscle mass, and anthropometric circumferences, in adults with and without training.

### 3.1. Body Mass

Of the 16 included studies, 13 assessed body mass. One of the earliest studies considering the effects of ASH on body composition was conducted by Cooley et al. [38]. Anxious adults lost more weight when undergoing naturopathic care, including 300 mg of ASH and a Senior Multi One multivitamin twice per day, compared with standardized psychotherapy with PLA supplements. Similarly, overweight and obese adults with high stress receiving 300 mg of KSM-66^®^ ASH root extract twice daily for 8 weeks had a 2.32 kg decrease in body mass and a 0.79 kg/m^2^ decrease in BMI [37]. These results were significantly greater than the 1.13 kg loss of body mass observed in PLA (*p* = 0.0148). Then, Watanabe et al. [48] used a double-blind, crossover design with four weeks per intervention and a two-week washout in overweight and obese adults. Participants received 150 mg of ASH and 300 mg of Chinese cinnamon (*Cinnamomum cassia*), or PLA, three times per day while consuming a hypocaloric diet at 20% less than their typical caloric intake. Those who received the supplement experienced a greater average 2.66% decrease in body mass compared to a 1.28% reduction in the PLA group (*p* = 0.0002). Those with obesity using the supplement lost a greater percentage of body mass at 3.8% compared to 2.8% in those who were overweight. Weight loss in both obese and overweight participants taking the supplement was significantly greater than PLA (−1.9%, *p* = 0.0143 and −1.9%, *p* = 0.009, respectively). When stratifying by intervention arm, there was a significant difference in relative body mass change between the groups during the first arm (*p* = 0.001), but not the second (*p* = 0.11) [48]. Recently, overweight and obese participants supplementing with 300 mg of KSM-66^®^ ASH root extract twice daily showed significant reductions in both body weight and BMI after 4, 12, and 24 weeks of supplementation compared to PLA (*p* < 0.0001) [41]. However, no other studies included reported changes in body mass or BMI. For example, Raut and colleagues [43] found no effect of 250 mg of Agewel^TM^ ASH root extract with 1.5% Withanolides, administered twice daily for 60 days, on BMI in young, healthy adults (*p* > 0.05).

### 3.2. Body Fat

#### 3.2.1. Fat Mass

Seven of the 16 included studies assessed total fat mass or body fat percentage. Another early study by Wankhede et al. [32] used 300 mg of KSM-66^®^ ASH root extract twice daily for 8 weeks in healthy men, with concurrent progressive resistance training. This study found a statistically significant 3.47% decrease in body fat in the ASH group (*p* < 0.001), as measured by bioelectrical impedance analysis (BIA), which was significantly greater than the 1.52% decrease in the PLA group (*p* = 0.03). However, no recent studies have reproduced these findings. For instance, in a study by Charmas et al. [36], healthy men taking part in a progressive high-intensity interval training (HIIT) program while supplementing twice per day with 300 mg of KSM-66^®^ ASH root extract had neither a change in nor a difference compared to PLA in percent fat mass (both *p* > 0.05).

#### 3.2.2. Waist Circumference

Four studies used waist circumference to assess changes in fat mass amongst overweight and obese participants. Most recently, Vaidya and colleagues [45] found that older adults who supplemented with 200 mg of ASH root extract twice daily for 6 months showed a significant reduction in waist circumference compared with PLA. Further, Ballesteros-Torres et al. [35] enrolled overweight and obese adults with normal blood glucose, high low-density lipoprotein cholesterol, total cholesterol, and triglycerides, and low high-density lipoprotein cholesterol. Participants received 500 mg of ASH root extract once daily, along with a personalized meal plan, for 40 days. Female participants showed a statistically significant between-group difference in waist circumference, with a 1.48 cm decrease in the ASH group versus a 0.97 cm increase in the PLA group (*p* = 0.0495). Male participants showed no difference between groups (*p* = 0.2752) [35]. In the crossover study by Watanabe et al. [48], when comparing the first arm, the ASH and *Cinnamomum cassia* group showed a statistically significant 2.75 cm decrease in waist circumference compared with a 1.73 cm decrease in the PLA group (*p* = 0.0307). However, this between-group significance was lost during the second arm (*p* = 0.239). When stratified by sex, after the first arm, males in the ASH group had a statistically significantly greater decrease in waist circumference (−3.14 cm) than in the PLA group (−0.70 cm; *p* = 0.0108). Females showed no significant difference between groups: the ASH group lost 2.58 cm, and the PLA group decreased by 2.10 cm (*p* = 0.3168). There was no difference in waist circumference between groups after the second arm when stratified by sex (both *p* = 0.3863) [48]. Finally, Smith et al. [44] found no effect of Witholytin^®^ supplementation with 1.5% Withanolides on waist circumference or waist-to-hip ratio when stratified by sex (all *p* > 0.05).

### 3.3. Muscle-Related Outcomes

#### 3.3.1. Fat-Free Mass, Lean Mass, and Muscle Mass

Only one study evaluated the effects of ASH supplementation on muscle mass without training. Pérez-Piñero et al. [42] compared four conditions in healthy adults with sleep problems: two doses of ASH (250 mg and 600 mg), 250 mg of ASH plus 175 mg of tryptophan, and a PLA. This study found no statistically significant change in muscle mass measured by BIA across groups, nor any between-group differences (all *p* > 0.05). Three other studies examined ASH’s effect on lean tissue, fat-free mass, or muscle mass across different exercise training interventions. Ziegenfuss et al. [49] showed no effect of 12 weeks of 500 mg of Sensoril^®^ with 10% Withanolides ASH root and leaf extract with progressive resistance training in men on lean mass or on lean/fat mass, measured by Dual-Energy X-ray Absorptiometry (DEXA), compared to PLA (*p* = 0.49 and *p* = 0.09, respectively). Similarly, Długołęcka et al. [40] and Charmas et al. [36] found no effect of twice daily 300 mg of KSM-66^®^ ASH root extract on fat-free mass in professional wrestlers or muscle mass in men taking part in HIIT, respectively (both *p* > 0.05).

#### 3.3.2. Anthropometric Measures of Muscle Size

Two other training studies used anthropometric measures to assess changes in muscle size during resistance training with ASH supplementation. The study by Wankhede et al. [32] showed significant between-group differences in arm and chest size. Arm size increased by 8.89 cm^2^ in the ASH group and by 5.30 cm^2^ in the PLA group (*p* = 0.01). For chest circumference, there was a 3.37 cm increase in the ASH group versus 1.43 cm in the PLA group (*p* = 0.0002). Additionally, while both groups significantly increased thigh size (*p* < 0.01), there was no significant difference between groups (*p* = 0.36) [32]. In an 8-week study, Verma et al. [47] supplemented healthy adults with 300 mg of KSM-66^®^ ASH root extract twice daily. The ASH group showed a significant overall increase in mid-arm circumference compared with PLA (*p* = 0.002), as well as a significant increase in chest circumference (*p* = 0.014). There was no significant overall difference in the change in thigh circumference between the groups (*p* = 0.519). When stratifying by sex, there was a significant difference in mid-arm circumference between groups in males (*p* = 0.009), but not in females (*p* = 0.226), and there was a significant difference in both chest (*p* = 0.006) and thigh circumference between the groups in females (*p* = 0.009), but not in males (both *p* > 0.05). However, baseline differences between groups in male, female, and total chest circumference (*p* = 0.019, 0.031, and 0.002, respectively), as well as female (*p* = 0.063) and total mid-arm circumference (*p* = 0.029), were also observed [47].

### 3.4. Other Effects on Body Composition

Other body composition metrics that were measured with ASH and various training interventions include android/gynoid fat ratio, visceral adipose tissue (VAT), total bone mineral content, and total body water. First, in the study by Ziegenfuss et al. [49], there was a significant between-group difference in android/gynoid ratio (*p* = 0.03); however, this was driven by a significant increase in the PLA group (*p* = 0.001), with no change in the ASH group (*p* = 0.99). Additionally, they found no effect of the intervention on VAT (*p* = 0.99). Similarly, Charmas et al. [36] found no changes or between-group differences in total body water after an ASH and HIIT intervention (*p* > 0.05). Finally, in a study by Długołęcka et al. [40], professional wrestlers supplemented with 300 mg of KSM-66^®^ ASH root extract twice daily for 8 weeks during the preparation period for the Polish Senior Championships. This study showed no effects on total bone mineral content in professional wrestlers (*p* > 0.05).

## 4. Discussion

### 4.1. Summary of Evidence

This review aimed to evaluate the effects of ASH supplementation on body composition. A synthesis of all major results from the current literature, with their respective experimental designs, is outlined in Table 1. While a recent meta-analysis showed no effects of ASH supplementation on body mass or body fat [50], analysis of the included studies found some effects on body mass, BMI, and fat mass, as well as sex-specific changes in waist, arm, chest, and thigh circumference compared to PLA. Significant changes in body mass and BMI were limited to 300 mg KSM-66^®^ twice daily in overweight and obese populations with and without a hypocaloric diet [36,40,47], with no effect in healthy adults with and without exercise intervention [36,42,43,45,46,47,49]. These findings suggest that ASH may have greater potential to influence body composition in adults with excess body mass. Between-study variation in population and intervention explains the lack of effect on body mass in the previous meta-analysis, which contrasts with the results measured in these studies [50]. Moreover, Watanabe et al. [48] found that those who were obese lost more percent body mass than participants who were overweight when eating a hypocaloric diet and supplementing with ASH and *Cinnamomum cassia*. Given the dietary restriction and cinnamon intervention, these results should be interpreted with caution, as weight loss cannot be attributed strictly to ASH supplementation. Additionally, Ballestero-Torres et al. [35] did not show an effect of ASH with diet monitoring on body mass in their overweight and obese population, indicating some variation in results.

Three of the four studies that measured waist circumference as a validated proxy for VAT [51] showed reductions in both healthy [45] and overweight and obese populations compared to PLA [35,48]. Interestingly, there were inconsistent impacts of sex on waist circumference between the studies by Ballesteros-Torres et al. [35] and Watanabe et al. [48], with one reporting a significant effect in females only and the other in males only when supplementing with dietary restriction. These contrasting results may be attributed to differences in supplement intervention or differences in caloric restriction, rather than ASH alone. Therefore, there may be an impact of ASH on waist circumference in overweight and obese adults, but more research is needed to fully dissect any sex-specific changes. However, Smith and colleagues [44] did not show an effect of supplementation without a diet intervention on waist circumference or BMI in either sex. None of these studies directly analyzed supplement x sex interactions, so these findings should be treated as exploratory and give rationale for future analysis into sex differences in response to ASH supplementation. Overall, it is possible that the different extracts and supplementation strategies used may have different impacts on changes in waist circumference, but inconsistencies in diet and population inhibit direct comparisons.

Studies measuring body fat percentage, by contrast, generally included healthy populations, in which only one [32] of the included studies measuring body fat found an effect of supplementation [36,40,42,43,47,49]. Given the potential effects of ASH on body mass and waist circumference in overweight and obese populations, it is possible that there would be greater and more consistent improvements in body fat percentage in these populations, compared to lower BMI groups. Furthermore, all three studies that analyzed body fat percentage using DEXA included an exercise training program but differed in their study populations. Both studies that did not find an effect of ASH on body fat used a recreationally active population [47,49], whereas the positive effect reported by Wankhede et al. [32] did not involve an active population. These results suggest that ASH supplementation may lead to greater fat loss in those initiating an exercise program than in those with a history of training. Two studies measured changes in anthropometric circumferences during resistance training as indicators of muscle size [32,47]. Both studies found a significant increase in overall arm and chest circumference but not in thigh circumference, compared with PLA. Anthropometric circumferences are a practical measure to estimate muscle size but are not rigorous enough to provide direct information about changes in overall lean tissue or muscle mass [52,53]. These results suggest that ASH supplementation alongside resistance training may improve muscle size, which would be advantageous for athletes seeking to increase it, such as bodybuilders [22], but more research using validated measures such as DEXA or BIA is needed to establish a causal relationship.

Nevertheless, many studies included in this review show no effect on body composition. For instance, no studies demonstrated improved lean mass, fat-free mass, muscle mass, central fat mass, bone mineral content, or total body water content [36,40,42,43,49]. The lack of increase in lean and muscle mass contrasts with the increases in arm and chest circumferences previously described. Measures of lean mass and muscle mass are obtained using either BIA or DEXA, which have greater validity for measuring muscle than circumference measurements [54,55,56,57,58]. Only one study assessed the variables VAT, android/gynoid ratio [49], bone mineral content [40], and total body water in response to training interventions [36]. Given the heterogeneity of the included studies, more research is needed to determine whether ASH affects any of these in any population.

### 4.2. Potential Mechanisms of Action

The most consistent findings from the included studies were improvements in body composition among overweight and obese individuals. There were significant reductions in both body mass and waist circumference in these populations [35,37,48]. A potential mechanism involves ASH’s regulation of the HPA axis. Stress increases HPA axis and sympathetic nervous system activity, resulting in the production of cortisol, adrenaline, and norepinephrine [4]. Chronic stress dysregulates HPA axis activity, leading to higher resting cortisol levels and an increased risk of illness [59]. ASH supplementation lowers cortisol levels, the body’s primary stress hormone. This regulation of cortisol leads to reductions in stress, depression, and anxiety, and improvements in insomnia symptoms [7]. Furthermore, this HPA axis regulation positively influences eating patterns. High HPA axis activity increases leptin production [60,61], decreases satiety, and increases consumption of high-fat and high-sugar foods [62]. Additional dietary fat, sugar, and overall caloric intake are associated with higher body fat and body mass [63]. By regulating hunger signals to reduce caloric intake by lowering cortisol, ASH may lead to lower body mass and body fat in those who overeat. This mechanism may explain why significant results were not observed for those with normal body weight, as they have regulated dietary intake.

There is further evidence of ASH’s improvements in body composition when considering the potential metabolic effects of the potent withanolide, withaferin A. One study detailed the improvements in glucose clearance, insulin sensitivity, glucose metabolism, and fat metabolism in obese mice on a high-fat diet treated with withaferin A [1]. First, withaferin A reduced leptin levels, resulting in lower food intake compared to untreated mice, consistent with the previously described mechanism. Low adiponectin levels are associated with increased adiposity and reduced insulin sensitivity in humans [64]. Normalizing adiponectin should increase insulin sensitivity and, therefore, glucose clearance. Next, treatment with this withanolide downregulated both glucose- and lipid-metabolism-related gene expression in the liver, including proliferator-activated receptors, compared with the dysregulation observed in untreated mice [1]. Similarly, in overweight and obese humans supplementing with ASH, there have been improvements in levels of triglycerides and very low-density lipoprotein cholesterol [35]. These preclinical findings provide a potential mechanistic rationale for the role of withaferin A in improving metabolism that requires confirmation in human studies. Finally, the mice had attenuated weight gain compared to mice without withaferin A treatment, indicating that the increased metabolism led to measurable changes in body mass [1]. People with higher body fat are more likely to develop metabolic syndrome and insulin resistance, which impair proper metabolic function [16,65]. Theoretically, this mechanism would improve metabolic function in humans, resulting in greater fat loss in persons with excess body fat or metabolic dysfunction. Therefore, ASH’s effects on improving glucose and fat metabolism may contribute to the changes in body and fat mass observed in the included studies, but preclinical results must be reproduced in humans before the mechanism is elucidated.

Results from clinical trials on the effects of ASH supplementation on testosterone concentration have been thoroughly summarized in previous reviews [8,66,67]. While the mechanism underlying the increase in testosterone with ASH supplementation remains uninvestigated, these influences on the HPA axis may affect testosterone production. Cortisol levels are inversely related to testosterone, meaning reductions in cortisol concentrations increase endogenous testosterone production [68]. Therefore, ASH supplementation may regulate the HPA axis, leading to higher testosterone levels. Additionally, in animal models, ASH exerts anti-inflammatory and antioxidant effects by suppressing NF-κB [25], thereby inducing the transcription factor Nrf2 and antioxidant gene expression [26]. Furthermore, ASH acts as an antioxidant by normalizing lipid peroxidation levels in arsenic-treated rats, thereby restoring reproductive function [69]. Since there is also an inverse relationship between testosterone and inflammation and oxidative stress [70,71], ASH’s ability to regulate these processes may increase testosterone levels. Though the preclinical mechanisms are promising, the measured increases in testosterone levels with ASH supplementation in humans do not translate into improved muscle mass with training. None of the included studies reported increased lean mass or muscle mass with ASH supplementation. ASH’s proposed indirect mechanisms do not appear to provide sufficient effect to yield additive benefits for muscle adaptation to exercise. However, differences in supplementation and exercise interventions across the included studies limit the ability to draw any distinct conclusions. More research is needed to elucidate the mechanism underlying ASH’s effects on testosterone and to determine how these hormonal responses can be leveraged to enhance athletic performance.

### 4.3. Recommendations for Future Research

Overall, more high-quality research on the effects of ASH on body composition is needed to draw definitive conclusions. Future research should prioritize the use of reliable body composition measures, such as DEXA, to improve measurement precision and comparability across studies. Based on the results of this review, the potential positive impacts of ASH on body composition in overweight and obese adults warrant further research to investigate the underlying mechanisms of weight loss in humans with ASH supplementation. Clarifying ASH’s mechanistic role in modulating the HPA axis for appetite and metabolism regulation would benefit those looking to lose weight. Future human studies should also implement both a diet and exercise program with supplementation in overweight and obese adults to observe potential additive benefits. Given the lack of evidence for effects on muscle mass in the current literature, more research is needed to understand how ASH’s impact on testosterone can be leveraged to improve overall body composition. The potential influence of sex on supplementation outcomes also warrants further investigation. Changes in circumference measurements differed between the sexes, indicating a sex effect on changes in body composition with ASH supplementation. These differences may reflect sex-specific hormonal profiles induced by supplementation; however, further research is needed to elucidate these potential mechanisms and assess direct sex-by-supplement interactions. Finally, standardizing the supplementation protocol should be a future priority to improve reproducibility, as each extract may not be pharmacologically interchangeable. Each extract uses different plant parts, extraction methods, extraction ratios, total withanolides, doses, and third-party analysis. Research should focus on optimizing ASH metabolites to improve body composition and other factors, such as mood regulation and performance adaptation, to develop the optimal ASH product.

## 5. Conclusions

The findings of this review revealed mixed results regarding the efficacy of ASH supplementation in improving body composition. Current evidence suggests that ASH is most beneficial for body mass and selected anthropometric outcomes in overweight and obese populations. Results in these populations may be improved with a healthy diet, increased exercise, and ASH supplementation. Evidence for reductions in fat mass and increases in lean mass remains inconsistent. Some studies indicate additive effects of ASH with strength training on muscle size, but more studies are needed to demonstrate an effect of ASH on lean mass, with potentially greater effects in those initiating an exercise program than in those with experience. Thus, there is rationale for future investigation into the effects of ASH on body composition, with a particular interest in interventions for individuals with excess body fat who are initiating an exercise program. More adequately powered randomized controlled trials are needed to establish an impact of ASH on body composition before recommending it for this purpose.

## Figures and Tables

**Table 1:** Evidence of the effects of ashwagandha supplementation on body composition.

Study	Design, Length	Population, Sex	Groups	Dose	Variables	ASH Change	PLA Change
Ballesteros-Torres et al. [35]	RDBPC with monitored diet, 40 days	Adults (BMI 25+, glucose < 100 mg/dL, LDL-c 160+ mg/dL, HDL-c < 40 mg/dL, trigly 150+ mg/dL, F and M	ASH = 21 PLA = 17	500 mg (2.5 mg/g Withanolides)	BM	−0.42 kg	1.14 kg
					BMI	−0.17 kg/m^2^	0.44 kg/m^2^
					WC	M: 0.9 cm F: −1.48 cm *	M: −5.53 cm F: 8.05 cm *
Charmas et al. [36]	RDBPC with progressive HIIT, 8 weeks	Young, healthy without regular PA, M	ASH = 18 PLA = 20	2 × 300 mg KSM-66^®^ (5% Withanolides)	BM	−0.1 kg	0.1 kg
					FM%	−0.6%	0.2%
					MM%	0.5%	0.1%
					TBW%	0.5%	0.1%
Choudhary et al. [37] ^⸸^	RDBPC, 8 weeks	Adults with chronic stress (BMI 25–39.9), F and M	ASH = 25 PLA = 25	2 × 300 mg KSM-66^®^ (5% Withanolides)	BM	−2.32 kg †*	−1.13 kg *
					BMI	−0.79 kg/m^2^ †*	−0.38 kg/m^2^ *
Cooley et al. [38]	RPC with stress-relief interventions, 6 weeks	Adults with anxiety without depression, F and M	ASH = 36 PLA = 39	Multi-ingredient: 2 × 300 mg (1.5% Withanolides) + Multivitamin	BM	−1.98 kg †*	−0.51 kg *
					BMI	−0.75 kg/m^2^ †*	−0.19 kg/m^2^ *
Coope et al. [39]	RDBPC with regular football training, 28 days	Young, healthy professional football players, F	ASH = 15 PLA = 15	600 mg KSM-66^®^ (5% Withanolides)	BM	0.1 kg	0 kg
Długołęcka et al. [40]	RDBPC with wrestling programming, 8 weeks	Young professional wrestlers, M	ASH = 10 PLA = 11	2 × 300 mg KSM-66^®^ (5% Withanolides	BM	0 kg	0.7 kg
					FM	−0.8 kg †	−0.5 kg †
					FFM	0.8 kg †	1.2 kg †
					MM	0.8 kg †	1.2 kg †
					Total BMC	−15.3 g	8.0 g
Pakhale et al. [41]	RDBPC, 24 weeks	Adults (BMI 25–39.9), F and M	A = 45 P = 46	2 × 300 mg KSM-66^®^ (5% Withanolides)	BM	−8.46 kg †*	−2.41 kg †*
					BMI	−3.31 kg/m^2^ †*	−0.93 kg/m^2^ †*
Pérez-Piñero et al. [42]	RDBPC, 90 days	Adults with poor sleep quality (BMI < 32), F and M	ASH 250 = 13 ASH 250 + TRP = 13 ASH 600 = 13 PLA = 13	Multi-ingredient: 250 mg, 250 mg + 175 mg TRP, 600 mg	BM	250: 0.3 kg 250 + TRP: 0.2 kg 600: 0.2 kg	0.3 kg
					BMI	250: −0.1 kg/m^2^ 250 + TRP: 0 kg/m^2^ 600: 0.1 kg/m^2^	0.1 kg/m^2^
					FM%	250: −0.1% 250 + TRP: −0.3% 600: 0%	−0.4%
					MM	250: −0.1 kg 250 + TRP: 0.3 kg 600: 0.2 kg	0.5 kg
Raut et al. [43]	RDBPC, 60 days	Young, healthy adults (BMI 18–30), 1 F and 51 M	ASH = 28 PLA = 24	2 × 250 mg Agewel^TM^ (1.5% Withanolides)	BMI	0.4 kg/m^2^	0.2 kg/m^2^
					FM%	−0.1%	1.2%
					MM%	1.01%	0.04%
Smith et al. [44] ^⸸^	RDBPC, 12 weeks	Older adults with fatigue and stress (BMI 25–35), F and M	ASH = 55 PLA = 56	2 × 400 mg Witholytin^®^ (1.5% Withanolides)	BMI	M: −0.24 kg/m^2^ F: −0.01 kg/m^2^	M: −0.14 kg/m^2^ F: 0.18 kg/m^2^
					WC	M: 0.53 cm F: −0.34 cm	M: −0.33 cm F: −3.06 cm
					WHR	M: 0 F: 0	M: 0 F: −0.3
Vaidya et al. [45] ^⸸^	RDBPC, 180 days	Older adults (BMI < 30), 4 F and 36 M	ASH = 20 PLA = 20	2 × 200 mg LongeFera™ (2.5% Withanolides)	BM	−0.52 kg	−0.33 kg
					BMI	−0.03 kg/m^2^	−0.05 kg/m^2^
					WC	−0.2 cm *	1.19 cm *
					HC	−0.9 cm *	0.85 cm *
Verma et al. [46]	RDBPC, 8 weeks	Young, healthy adults, F and M	ASH = 40 PLA = 40	2 × 300 mg KSM-66^®^ (5% Withanolides)	BM	1.29 kg	0.98 kg
					BMI	0.51 kg/m^2^	0.39 kg/m^2^
Verma et al. [47]	RDBPC with progressive resistance training, 8 weeks	Healthy adults with regular PA, F and M	ASH = 37 PLA = 36	2 × 300 mg KSM-66^®^ (5% Withanolides)	BM	1.3 kg †	1.0 kg †
					BMI	0.5 kg/m^2^ †	0.4 kg/m^2^ †
					FM%	−1.7% †	−1.7% †
					Arm C	Total: 1.5 cm †* F: 1.5 cm † M: 1.6 cm †*	Total: 1.2 cm †* F: 1.2 cm † M: 1.2 cm †*
					Chest C	Total: 3.6 cm †* F: 3.5 cm †* M: 3.7 cm †	Total: 2.5 cm †* F: 2.0 cm †* M: 2.9 cm †
					Thigh C	Total: 2.1 cm † F: 0.8 cm †* M: 3.4 cm †	1.8 cm † F: 0.6 cm †* M: 2.9 cm †
Wankhede et al. [32]	RDBPC with progressive resistance training, 8 weeks	Healthy adults, M	ASH = 25 PLA = 25	2 × 300 mg KSM-66^®^ (5% Withanolides)	FM%	−3.47% †*	−1.52% †*
					Chest C	3.37 cm †*	1.43 cm †*
					Arm size	8.89 cm^2^ †*	5.30 cm^2^ †*
					Thigh size	8.71 cm^2^ †	6.22 cm^2^ †
Watanabe et al. [48]	RDBPC crossover with hypocaloric diet, 4 weeks each arm	Overweight or obese adults (BMI 26–35), M and F	ASH = 22 PLA = 18	Multi-ingredient: 3 × 150 mg + 300 mg *Cinnamomum cassia*	BM	−2.66% * Obese: −3.8% * OW: −2.8% *	−1.28% * Obese: 1.9% * 0.9% *
					WC	Week 4: −2.75 cm * F: −2.58 cm M: −3.14 cm * Week 10: −1.39 cm F: −1.02 cm M: −2.28 cm	Week 4: −1.73 cm * F: −2.10 cm M: −0.70 cm * Week 10: −2.64 cm F: −2.46 cm M: −3.25 cm
Ziegenfuss et al. [49] ^⸸^	RDBPC with progressive resistance training, 12 weeks	Healthy, recreationally active adults (BMI 20–35), M	ASH = 19 PLA = 19	500 mg Sensoril^®^ (10% Withanolides)	BM	−0.6 kg	−0.6 kg
					FM%	−0.6%	0.2%
					FM	−0.6 kg	0.2 kg
					LM	0.4 kg	0.0 kg
					LM/FM	0.12	−0.9
					A/G	0 *	0.09 †*
					VAT	−0.02 kg	−0.03 kg

Data are expressed as change from baseline for the each group; † significant difference compared to baseline (*p* < 0.05); * significant difference between groups (*p* < 0.05); ⸸ studies sponsored by commercial companies; A/G: android/gynoid ratio; Agewel™: Standardized *Withania somnifera* (Ashwagandha) root extract (Pharmanza Herbal Pvt. Ltd., Gujarat, India); ASH: ashwagandha; BM: body mass (kg); BMC: bone mineral content (g); BMI: body mass index (kg/m^2^); C: circumference (cm); HC: Hip circumference; HIIT: high-intensity interval training; HDL-c: high-density lipoprotein cholesterol (mg/dL); F: female; FM: fat mass (kg or %); LDL-c: low-density lipoprotein cholesterol (mg/dL); LM: lean mass (kg); LongeFera™: Proprietary *Withania somnifera* (Ashwagandha) root extract (PhytoVeda Pvt. Ltd., Mumbai, Maharashtra, India); KSM-66^®^: Standardized *Withania somnifera* (Ashwagandha) root extract (Ixoreal Biomed Inc., Los Angeles, CA, USA / Hyderabad, India); M: male; MM: muscle mass (kg or %); OW: overweight: PLA: placebo; RDBPC: randomized double blind placebo control; Sensoril^®^: Standardized *Withania somnifera* (Ashwagandha) root and leaf extract (Natreon, Inc., New Brunswick, NJ, USA); TG: triglycerides (mg/dL); TBW: total body water (%); TRP: tryptophan; VAT: visceral adipose tissue (kg); WC: waist circumference (cm); WHR: waist-to-hip ratioWitholytin^®^: Standardized *Withania somnifera* (Ashwagandha) root extract (Verdure Sciences, Inc., Noblesville, IN, USA).

## Data Availability

No new data were created or analyzed in this study. Data sharing is not applicable to this article.

## Abbreviations

The following abbreviations are used in this manuscript:

ASH	Ashwagandha
HPA	Hypothalamus–pituitary–adrenal
CDC	Centers for Disease Control and Prevention
BMI	Body Mass Index
NF-κB	Nuclear Factor-kappa B
Nrf2	Nuclear factor erythroid 2-like 2
DHEA-S	Dehydroepiandrosterone sulfate
PLA	Placebo
BIA	Bioelectrical impedance analysis
HIIT	High-intensity interval training
DEXA	Dual-Energy X-ray Absorptiometry
VAT	Visceral Adipose Tissue
